# Barriers and Motivators for Referral of Patients with Suspected Lynch Syndrome to Cancer Genetic Services: A Qualitative Study

**DOI:** 10.3390/jpm4010020

**Published:** 2014-02-18

**Authors:** Yen Y. Tan, Lisa J. Fitzgerald

**Affiliations:** 1 School of Medicine, The University of Queensland, 288 Herston Road, Herston, QLD 4006, Australia; 2 Molecular Cancer Epidemiology Laboratory, Genetics and Computational Biology Division, QIMR Berghofer Medical Research Institute, 300 Herston Road, Herston, QLD 4006, Australia; 3 Queensland Centre for Gynaecological Cancer Research, Level 6 Ned Hanlon Building, Royal Brisbane and Women’s Hospital, Butterfield Street, Herston, QLD 4029, Australia; 4 School of Population Health, The University of Queensland, 288 Herston Road, Herston, QLD 4006, Australia; E-Mail: l.fitzgerald@sph.uq.edu.au

**Keywords:** Lynch syndrome, clinical genetics, referral, barriers and motivators knowledge, attitudes and referral practice

## Abstract

This article explores the views of general practitioners and specialists on their referral of patients with suspected Lynch syndrome to cancer genetic services. Using a purposive maximum variation sampling strategy, we conducted semi-structured interviews face-to-face with 28 general practitioners and specialists in public or private hospitals and specialist clinics between March and August 2011. General practitioners and specialists were recruited in a major metropolitan area in Australia. Interview transcripts were reviewed by two independent researchers, and thematic analysis was performed using NVivo10 software. The main barriers and motivators identified were: (1) clinician-related (e.g., familiarity with Lynch syndrome and family history knowledge); (2) patient-related (e.g., patients’ interests and personal experience with cancer); and (3) organizational-related (e.g., access to services, guidelines and referral pathway). Referral of patients with suspected Lynch syndrome to cancer genetic services is motivated and hindered by a range of individual, interpersonal and organizational factors. In order to improve the care and quality of life of patients and family with suspected Lynch syndrome, further research is needed to develop supportive tools for clinicians.

## 1. Introduction

Lynch syndrome, also known as hereditary non-polyposis colorectal cancer (HNPCC), is an autosomal dominantly inherited condition caused by germline mutations in one of the mismatch repair (MMR) genes *MLH1*, *MSH2*, *MSH6* and *PMS2*. It is estimated that about 2%-5% of all diagnosed colorectal and endometrial cancers are due to Lynch syndrome [1]. Compared to the general population, Lynch syndrome mutation carriers have a lifetime risk of up to 70% of developing colorectal cancer [2]. Women with germline mutation have an additional lifetime risks of up to 70% of developing endometrial cancer [2,3], and are also at a significantly increased risk of colorectal, kidney, renal pelvis, ureter, urinary bladder and breast cancer ten years following endometrial cancer diagnosis [4].

Given the substantial risk of cancers in Lynch syndrome individuals, identification of individuals and family members at increased risk of developing cancer for genetics services (*i.e.*, genetic counselling, risk assessment and/or genetic testing) is therefore important. Once a mutation carrier is identified, regular colonoscopy surveillance or risk-reducing surgeries can be initiated to reduce cancer risks [5,6,7]. However, Lynch syndrome is often under-recognized, even when patients have clear criteria unrelated to family history [8]. General practitioners or medical specialists often feel they lack sufficient knowledge to select individuals eligible for genetics services [9,10,11,12,13,14,15,16], and lack time or skills to collect adequate family history information [17,18,19,20,21,22]. It is also known that other factors such as referral guidelines and awareness of genetics services as well as patients requests affect clinicians’ decision to refer patients for genetics consultation [15,20,22]. Most of these studies, however, were conducted in North America and Europe, and on hereditary breast-ovarian cancer patient population.

In Australia, there is little known about the processes and practices of referral for cancer genetics services, particularly for patients suspected to have Lynch syndrome. Although most of genetics services are covered by Medicare (Australian public health insurance scheme) and are available to eligible patients referred for genetic consultation and/or testing, only a small proportion of patients suspected to have hereditary colorectal and endometrial cancer were referred for cancer genetics services [19,23]. Thus, the objective of this paper is to explore and identify barriers and motivators of referral for patients suspected to have Lynch syndrome for cancer genetics services. This study is part of a larger study, known as the eMBRACE Study, which aimed to improve identification and referral of patients with suspected Lynch syndrome for genetic services in Australia.

## 2. Experimental

This study was approved by the Human Research Ethics Committee of Royal Brisbane and Women’s Hospital, and the University of Queensland. We conducted 28 semi-structured interviews face-to-face with general practitioners and specialists in public or private hospitals or specialist clinics to investigate the barriers and motivators for referring patients with suspected Lynch syndrome to cancer genetics specialists.

### 2.1. Participants and Recruitment

Using data available within the public domain and the Australian Medical Association database, a random sample of 253 general practitioners and medical specialists involved in the treatment and follow-up care for women with or at increased risk of developing endometrial, ovarian and/or colorectal cancer, aged between 22 to 80 years, and practicing within 20 km of Brisbane city were sent a letter inviting their participation. Specialists from gynaecology, gastroenterology, medical oncology, radiation oncology, gynaecology oncology, and general and colorectal surgeons were included in the sample.

All potential participants were approached directly via post. Email was sent (*n* = 3) only if no postal address was available on the public domain and the Australian Medical Association database. Snowball sampling was used to identify additional participants practicing at Royal Brisbane and Women’s Hospital—a major tertiary public hospital in Queensland, Australia. All potential participants were provided with a study information brochure and a response form to return to the research team. Once the participants agreed to participate in the study, they were followed up by a phone call or email to arrange for an interview appointment. Non-responders were followed up with two reminder letters and a final notice, as per Dillman’s survey protocol [24]. In all, 62 (25%) responded, and 40 (16%) agreed to participate. Using a purposive maximum variation sampling strategy (*i.e.*, selecting participants from each specialty to ensure maximum diversity), we selected 28 participants and interviewed each one face-to-face in their practice room between March and August 2011.

### 2.2. Data Collection

Demographic data such as gender, specialty, type and location of practice were collected at the beginning of the interview. The interviewer then re-iterated the nature of the study and gave a broad outline of the interview content, and obtained informed consent from participants. All interviews were conducted by one investigator (YT) based on a standard interview guide developed around a core set of topics (see Table S1), including: (1) clinicians’ perceived barriers and motivators for referral of patients with suspected Lynch syndrome to cancer genetics services; (2) clinicians’ knowledge about Lynch syndrome; and (3) clinicians’ attitudes toward genetic testing. The topics were explored with each participant, and probes were used as needed to elicit greater detail dependent on each participant’s response and specialty. Interviews continued until no more new insights emerged (concept of thematic saturation). All interviews averaged about 30 min in length and were audio-recorded with permission.

### 2.3. Data Analysis

Data was managed and analysed using NVivo10 qualitative analysis software (QSR International Pty Ltd., Melbourne, Australia). Each interview was transcribed verbatim, and was de-identified to ensure confidentiality. Thematic analysis was carried out using methods as described by Braun and Clarke [25] with the aim to identify recurring themes and patterns in participants’ perceptions of barriers and motivators of referral. Two independent researchers (YYT and LJF) reviewed the transcripts and searched for initial concepts and emerging themes. Themes such as knowledge, attitudes and practice were coded, reorganized and refined to identify sub-themes, and were subsequently classified into the concepts of barriers and motivators. Barriers and motivators were then classified into physician-, patient- or organisational-related factors. For presentation of results, codes were designated for each participant according to their specialty *i.e.*, GP, general practitioners; GYN, gynaecologists; GO, gynaecological oncologists; MO, medical oncologists; GE, gastroenterologists.

## 3. Results

A summary of participant characteristics was shown in Table 1. All clinicians except two gynaecologists have referred patients for some sort of genetic consultation. We extracted from the interviews factors, which according to the clinicians influence their decision to refer patients with suspected Lynch syndrome for cancer genetics services. A summary of key findings is presented in Table 2. Extended quotes for both barriers to and motivators for genetics referral are presented in supplementary tables (Tables S2 and S3).

**Table 1:** Participant characteristics.

Specialty	Male, n	Female, n
General practitioners	1	6
Gynaecologists	5	1
Gynaecology oncologists	5	1
Medical oncologists	1	2
Gastroenterologists	5	1
**Total**	**17**	**11**

Three broad categories were identified: (1) clinician-related factors; (2) patient-related factors; and (3) organizational-related factors.

### 3.1. Barriers to Referral

#### 3.1.1. Clinician-Related Factors

##### 3.1.1.1. Lack Familiarity or Knowledge of Lynch Syndrome

Gastroenterologists and oncologists who participated in the study were more aware of Lynch syndrome than general practitioners and gynaecologists. The latter group of clinicians were confused between the term Lynch syndrome and HNPCC, but were more familiar with the latter term and its association with colorectal cancer.

*“I didn’t know anything about Lynch syndrome. I know breast cancer (BRCA) genes in breast cancer (are) important at determining your probability to develop breast cancer but I don’t know about any genes in endometrial cancer or bowel cancer.”*

*[GP21]*

**Table 2:** Summary of barriers and motivators for genetics referral of patients with suspected Lynch syndrome.

Factors	Barriers	Motivators
Clinician-related	Lack familiarity with Lynch syndrome	Knowledge of family history and age at diagnosis
	Lack of adherence to guidelines	Knowledge of tumour test results
	Negative attitude toward genetic testing	Improvement for patient diagnosis, treatment and clinical management
	Lack of professional experience	
	Uncertain of who or when to refer	
	Lack of awareness to importance of family history	
Patient-related	Patients disinterest	Patients requests
	Lack of family history knowledge to guide referral	
Organizational-related	Uncertain or perceived long wait time for a genetics appointment or test results	Practical information about genetic services (e.g., the availability and cost of testing, turnaround time)
	Unknown cost or assumed high costs of testing	Specific criteria or guidelines for referral
	Unfamiliar with genetic services	Increased collaboration with genetics specialist
		Prompts or triggers for referral
		Ease of access for services
		Continuing education for clinicians
		Better follow-up care or referral pathway

*“I get confused between Lynch 1 and Lynch 2, and what sorts of cancers are involved.”*

*[GYN4]*

When clinicians were asked about the endometrial cancer risk in women with suspected Lynch syndrome, clinicians consistently under-recognized or were uncertain about the risk.

*“I’m not a geneticist and I couldn’t give you numbers (risk estimates).”*

*[GP6]*

*“I think it (endometrial cancer) rates after colon in terms of risk.”*

*[GE27]*

##### 3.1.1.2. Lack of Adherence to Guidelines

Some general practitioners were unaware of clinical guidelines for Lynch syndrome. Those who were aware generally did not find guidelines helpful as they lacked specificity for Lynch syndrome.

*“If there’s been family history that’s really been my only guideline.”*

*[GP28]*

*“Where to find these guideline is a problem, change of guideline is a problem...and the accessibility and the accountability as well who is going to be responsible if you don’t follow the guideline?”*

*[GYN12]*

*“In my experience, written guidelines…don’t work because nobody’s got time to read them.”*

*[GE20]*

##### 3.1.1.3. Negative Attitude toward Genetic Testing

About one-third of the clinicians had negative perceptions towards cancer genetic testing. Most raised concerns about the clinical utility of genetic test results, and concerns about insurance coverage of services. While one gynaecologist thought genetic testing would not alter clinical management or treatment for their patients, another gynaecologist perceived genetic counselling as preventive intervention and is secondary to treatment. Several clinicians considered family history collection to be time consuming.

*“There is a limitation on how useful testing is…how is this going to affect this person’s life? Is it going to add quality of life or quantity of life?”*

*[GP11]*

*“(Genetic testing) potentially opens cans of worms as far as the daughter’s insurability is concerned…knowing that she’s got the genetic predisposition to cancer doesn’t change anything…”*

*[GYN10]*

*“Knowing this genetic information have some potentially insurance implications…it has implications for other family members who may or may not want to know.”*

*[GO9]*

##### 3.1.1.4. Lack of Personal and Professional Experience

Many clinicians acknowledged that Lynch syndrome is a rare disorder and that they had little contact with patients with such syndrome.

*Lynch syndrome you can probably count on one hand...or probably a couple of fingers per year.”*

*[GE27]*

*“I haven’t come across anyone in my practice, it’s a rare thing.”*

*[GYN4]*

Other reported barriers include uncertainties of who or when to refer as well as lack of awareness of importance of family history.

#### 3.1.2. Patient-Related Factors

##### 3.1.2.1. Patients Disinterest

Referral to genetic specialists was highly dependent on patients’ willingness to attend genetics appointment. Although clinicians felt that their role was to provide information to patients, they stated that it was ultimately the patient’s choice to proceed with any surgeries, treatments or procedures.

*“I can make a referral and patients won’t go if they don’t want to.”*

*[GP6]*

*“My job is I guess to provide the information and then the patient can decide what they want…”*

*[GO9]*

*“Some patients don’t want to go there you know they’re stressed or you refer them they don’t turn up…”*

*[MO17]*

Participants described how most of their patients who declined genetics referral were worried about the cost of testing and the negative implications of test results for themselves and their family. While some patients did not think genetic testing would alter screening or treatment for themselves or their family members, some were afraid to know if they had inherited the genetic mutation.

*“People get worried about cost of genetic testing because it’s quite expensive a lot of these tests are expensive…”*

*[GO18]*

*“That* *has* *been brought up by one patient they were worried about it getting the testing done with the repercussions for their children.”*

*[GO8]*

##### 3.1.2.2. Lack of Family History Knowledge to Guide Referral

Some clinicians found it difficult to elicit sufficient family history information from patients, especially for those who did not know the medical history of their family.

*“…no patients know their full family history until they go asking sometimes you got to trigger them to go back to the family and ask for them what the broader family history is”*

*[GE14]*

#### 3.1.3. Organizational-Related Factors

##### 3.1.3.1. Uncertain Wait Times and Cost of Services

Many clinicians were uncertain about the wait time and cost of genetics services; some clinicians assumed that a genetics referral and testing would be very expensive for their patients. Hence, many were very concerned about the reimbursements and coverage of genetics services for their patients from both private health insurance and Medicare.

*“I don’t* *know how easy it is to get into clinical geneticists maybe some hideous waiting time…of years.”*

*[GYN10]*

*“Lot(s) of it is not covered by Medicare so patients do end up out of pocket so that can be a barrier.”*

*[GP11]*

*“I don’t* *know what contribution private health insurance would make to the payment. I don’t know where the covers are.”*

*[GO2]*

##### 3.1.3.2. Unfamiliar with Genetic Services

Clinicians in general were unfamiliar with cancer genetics services. They were either unaware of the availability of the service, or were uncertain of where and how to access such service when needed. They usually found out information for referral by word-of-mouth recommendations. Clinicians also reported their unfamiliarity with the type of services available, the quality and quantity of services provided, and the type of investigations needed to refer patients. Only those who had referred patients were aware of the organization and the availability of genetics services.

*“No I’ve never* *referredanyone like that. I never knew that this service exists.”*

*[GYN15]*

*“When I first came to town I don’t know where to send them…I didn’t know there was a service until someone said…somehow I’d sent one.*

*[GE16]*

*“I can’t find the cancer care website half the time, (and) I work here”*

*[MO17]*

*“I don’tknow what investigations the genetics people would want...”*

*[GO18]*

### 3.2. Motivators for Referral

#### 3.2.1. Clinician-Related Factors

##### Knowledge of Family History, Age at Diagnosis and Tumour Test Results

All clinicians considered that a significant family history of cancer and young age at cancer diagnosis were strong indicators for referral. Some specialists were more inclined to recommend patients for genetics services if tumour testing, such as microsatellite instability or immunohistochemistry, were conducted to screen for evidence of mismatch repair deficiency.

*“If they have mismatch repair (testing) then I’ll be more inclined to refer if there’s no mismatch repair then the chances are that I’d probably won’t refer because it’s an isolated case.”*

*[MO5]*

*“if a genes is being identified in your family and you can be tested in one way or the other then I think that opportunity needs to be given to everyone.”*

*[GP11]*

Most clinicians described genetic testing as very useful to improve diagnosis for patients, and for prevention and treatment for both patients and family members.

#### 3.2.2. Patient-Related Factors

##### Patients Requests

Most of the clinicians recommended genetics consultation for patients with suspected Lynch syndrome. It was uncommon for patients to request genetic testing. However, patients with a personal or family history of cancer were more likely to request such services, and so were mothers’ who were concerned about their daughters’ risk of cancer.

*“…slightly more common for us to suggest it (referral). Often those women would have concerns for their children and they want to be tested for that reason.”*

*[GP11]*

#### 3.2.3. Organizational-Related Factors

##### 3.2.3.1. Practical Information about Genetic Services

General practitioners and specialists described how they would like clarification of the role of clinical genetics and more information on the referral pathway (*i.e.*, who is appropriate to refer, when to refer and how to refer). Some general practitioners wanted succinct and practical information about the availability of the tests, the cost of testing and the turnaround time as such information would assist patient consultation, and subsequently result in the uptake of referral.

*“what would be the criteria that they want to see patients referred but also a very brief dot points of the things that they can offer… advice with regards to life insurance and family planning… the key roles of clinical geneticists and then guidelines for referral for consideration of Lynch or other syndromes would be useful.”*

*[GE25]*

*“In general practice really you just want the practical stuff… accurate information on the availability and cost of testing is useful…”*

*[GP11]*

Two general practitioners requested Lynch syndrome information brochure for their patients to help patients to understand the implications of genetic testing and the referral process involved. One gynaecologist and one general practitioner also wanted more information about the possibility of requesting for additional tests to a previous pathological sample, and the type of information to be gathered by patients prior to genetics attendance.

##### 3.2.3.2. Specific Criteria for Referral and Increased Collaboration with Genetics Specialists

Most clinicians considered guidelines to be helpful in guiding appropriate referral. A few general practitioners who had previously referred patients for both private and public genetics services indicated the need for clarification of the threshold for referral, and wanted more specific guidelines for patients suspected with Lynch syndrome.

*“Guidelines would be good. We have Amsterdam criteria but it’d be nice if there was Australia wide guidelines-that would be ideal.”*

*[GO7]*

*“I think having clearer guidelines from genetics might be helpful such as when to refer... it’ll be ideal if they have them on a board in the multidisciplinary room.”*

*[MO17]*

Oncologists and gynaecologists expressed their interests for increased collaboration with the genetics specialists to improve genetic literacy and awareness of genetics services and available resources.

##### 3.2.3.3. Prompts or Triggers for Referral

Prompts for referral, such as an online tool for risk assessment with an integrated appointment booking system, were suggested to ease identification and referral of the patients at a general practice clinic. A central database and a coordinator were considered important for follow-up care management of patients.

*“I don’t know whether in some ways (a) clinical software…that you can flag everyone that’s got a family history of breast cancer for instance and then pull them up and… send them a brochure on genetics in breast cancer...”*

*[GP21]*

*“…more important than the referral to the geneticists is sometimes the kind of coordination of the you know prevention and management”*

*[MO22]*

##### 3.2.3.4. Ease of Access

The gynaecologists, gastroenterologists, and oncologists who had previously referred patients reported ease of access to clinical genetics services. They knew where and who to contact when genetics services was warranted, and were content that an appointment could be organized efficiently through a single phone call.

*“I just ring them and say…I need the patients to be on the list and (they) say okay well this is the date and time the patient can come and see me.”*

*[GYN13]*

*“It’s not a big deal for me it’s just a phone call”*

*[GE3]*

Few clinicians reported the need for continuing professional education to heighten awareness of Lynch syndrome. Some other clinicians suggested a more comprehensive follow-up pathway for patients with suspected Lynch syndrome.

## 4. Discussion

The findings presented here provide an insight into the referral practices of a range of clinicians. To the best of our knowledge, this is the first study to explore in detail the view of clinicians about referring patients suspected to have Lynch syndrome for cancer genetics services. Australian clinicians were generally positive about referring patients for cancer genetics services, but lack knowledge and support in which they need to make an appropriate referral. Our results support the outcomes of previous studies regarding deficiency in knowledge and awareness about Lynch syndrome and Lynch-associated tumour types [19,20]. The syndrome’s complexity, which involved not only colorectal cancer aggregation in a family but also malignancies in the endometrium, ovary, stomach, small bowel, pancreas, liver and biliary tract, upper urinary epithelial tract, skin, brain and possibly breast, may serve as confounders for diagnosing high-risk families [26]. In addition, the inconsistent nomenclature used for the syndrome can lead to poor recognition and referral of appropriate patients who would benefit from cancer genetics services. There was general agreement that increased continuing education would improve knowledge and awareness of Lynch syndrome. General practitioners and specialists wanted educational information materials about Lynch syndrome, including the indicators for referral, the types of services available, as well as advice regarding cost, waiting time, life insurance and family planning from the genetics services. Such information would serve as reference during patient consultations, and also a reminder to refer patients suspected to have Lynch syndrome for cancer genetics services. Nevertheless, the development of such information material needs to consider change over time, and would require further assessment for its effectiveness in both general and specialist practice settings. Future research may employ pre-test, exposure and post-test or longitudinal designs to more definitely assess clinicians’ knowledge and their referral practice.

The clinicians interviewed in the study wanted information brochures for patients with increased risk of Lynch syndrome to help inform and persuade high-risk patients and their family to attend the recommended genetics consultation as well as increase patient motivation and adherence to screening and treatment. One recent study has shown that written information has effectively increases patients overall understanding of disease and prompts for family communication and test uptake [27]. However, participants were not aware that such patient information has been developed in Australia and is available through the Centre for Genetics Education [28]. Widespread dissemination of information to general and specialist practitioners is needed, and further studies are necessary to determine whether written information increases patients’ adherence to recommended treatment or screening regimens.

Family history of cancer is an important factor for referral. However, many interviewees in the study reported lack of both time and patients’ family history knowledge. Findings from other studies suggest that Lynch syndrome is under-recognized because family history is infrequently obtained [8,18,19,29,30,31], and lack detail when family history is collected [21,30,32,33,34]. Since time constraints are considered an obstacle for family history collection in clinical practice, self-administered family history forms and computer-based family history tools have been developed and have been proven to be effective and convenient for collecting family history [19,35]. However, the methods for family history collection have varied across studies [36], and would therefore benefit from standardized family history questions.

Published diagnostic guidelines for Lynch syndrome, such as the Amsterdam criteria or the Bethesda guidelines were developed to aid identification of possible Lynch syndrome patients based on patient’s personal and family history of cancer and tumour characteristics. However, the clinicians interviewed wanted simpler indications for referral, suggesting the possibility that some clinicians were not familiar with the published guidelines, and therefore need more support in the risk assessment process to identify appropriate patients for referral. The most commonly mentioned approach was simply to have prompts for referral when patients present with family history of cancer indicative of Lynch syndrome. Thus, a computerized family history and risk assessment tool based on recognized guidelines with built-in decision support for referral and easy online appointment booking system may facilitate identification of possible Lynch syndrome patients, and improve access to genetic consultation. The integration of such a tool, including information about tumour testing, cost of testing and reimbursements, and guides to follow-up care into the electronic health system would help clinicians overcome anxiety about their lack of expertise and increase confidence in delivering genetic health care. However, implementation of such a system is complicated [37], and must be studied, evaluated and adapted to ensure the suitability of use in both general and specialty practice settings.

The clinicians interviewed described how increased liaison between genetics and non-genetics specialists through conferences, in-house meetings and multidisciplinary team meetings (MDTs) would increase awareness of cancer genetics services, and improve communication flow between all those involved in the treatment of patients. The introduction of general practitioners and/or genetics specialists into the regular MDTs is therefore likely to simplify referral process and allow for collaborative consultation that facilitate efficient treatment planning and care management. Previous studies have shown that care coordination through MDTs is associated with decreased time from diagnosis to treatment [38], more accurate pathological staging of cancer [39], and improved patient survival [40]. However, the widespread adoption of MDTs in cancer care needs to be further evaluated for its performance and cost-effectiveness. Further research should explore the characteristics of clinicians involved in multidisciplinary care and how they work together as clinical teams to provide optimal care for patients. There is also a need to explore the patients’ experience and care support provided by the MDTs, and their adherence to the prescribed treatment or screening.

There are several factors that may limit the generalizability of findings from this study. First, the sample may not be representative of all clinicians who are involved in the treatment and follow-up care of women with suspected Lynch syndrome. Participants who agreed to be interviewed were urban practitioners, and were likely to be more interested in cancer genetics. Rural practitioners may have different views toward referrals of patients with suspected Lynch syndrome. Hence, a larger study is needed for generalizability of results; Secondly, the response rate was low; however, this is not unusual for health professions [41]. There was a general lack of interest and participation among surgeons and radiation oncologists—only 1 surgeon and 1 radiation oncologist responded to the invite with interest, but was unavailable for interview appointment. As such, surgeons and radiation oncologists may have different views regarding referring patients with suspected Lynch syndrome for genetics services. The unique regional health care systems and policies may also have influenced patients’ uptake of genetic consultation and/or testing.

## 5. Conclusions

Referral to cancer genetics services is motivated and hindered by a range of individual, interpersonal and organizational-related factors. We hope that the issues highlighted in this study will assist in improving education and awareness of Lynch syndrome at the clinicians (and patients) level, and the commitment of resources to support the provision of genetics services. Finally, we hope that our findings will guide the development of future initiatives to improve genetic health care delivery, leading to positive behaviour changes and better health outcomes for patients.

## Supplementary Files

Supplementary File 1 Supplementary Information (PDF, 659 KB)
