# Supplementary material for: Barriers and Motivators for Referral of Patients with Suspected Lynch Syndrome to Cancer Genetic Services: A Qualitative Study
Source: J Pers Med. 2014 Feb 18;4(1):20–34. doi: 10.3390/jpm4010020 (PMC4251408; doi:10.3390/jpm4010020)
Supplement: Supplementary File 1 — Supplementary Information (PDF, 659 KB) [file jpm-04-00020-s001.pdf]

Supplementary Information for

Barriers and Motivators for Referral of Patients with Suspected Lynch Syndrome to Cancer Genetic Services: A Qualitative Study

Table S1. Interview guide.

Themes	Question items
**About the clinicians**	1. Can you tell me about what you do in your practice?<br>2. How many years have you been practicing?<br>3. How many patients do you see per week?<br>4. How many are cancer patients?
**Referral of patients with suspected Lynch syndrome**	1. Have you encountered any patients that requested for referral for genetic counselling and testing?<br>2. Why did you refer the patients? What are the motivational factors for referring the patients?<br>3. Where did you refer them to? e.g., specialist clinic or pathology testing?<br>4. What are the barriers and motivators?
**Clinicians knowledge of Lynch syndrome**	**Set A (for those who had encountered or referred patients):**<br>1. What do you think the risk of cancer for patients with Lynch syndrome?<br>2. What do you think of endometrial cancer risk for Lynch syndrome patients?<br>**Set B (a patient scenario relevant to Lynch syndrome was presented for those who has never referred patients with suspected Lynch syndrome):**<br>1. What would you advice your patient of her genetic risk? What hereditary syndrome do you think this is?<br>2. Are there any diagnostic or molecular tests for this syndrome?
**Clinicians attitudes toward genetic testing**	1. How do you feel about genetic testing?<br>2. How do you perceive your role in relation to identifying these patients?<br>3. Who do you think should play the biggest role in identifying and referring these patients?
**About improving genetic services for patients**	1. What might help you to refer eligible patients? Information sheet? Guidelines?<br>2. How would you like things to be improved?<br>3. Is there anything else you would like to add?

Table S2. Barriers to referral of patients with suspected Lynch syndrome for cancer genetics services.

Thematic category	Barriers	Direct quotes
**Clinician-related factors**	Lack familiarity or knowledge of Lynch syndrome	*"So most people know what HNPCC is but Lynch syndrome is an older expression because of its discovery so…I guess we just need to make sure everyone's on the same page" [MO22]*<br>*"I didn't know anything about Lynch syndrome…I know… BRCA genes in breast cancer (are) important at determining your probability to develop breast cancer but I don't know about any genes in endometrial cancer or bowel cancer." [GP21]*<br>*"I get confused between Lynch 1 and Lynch 2 and... what sort of cancers are involved… and the hereditary polyposis is another one…that's usually endometrial and colon and… ovarian component to that one as well and there's couple of other ones thrown in." [GYN4]*<br>*"I don't think many people know what Lynch is, I've got to say, I don't think that's a common one but breast cancer gene is reasonably well" [GO18]*<br>*"I don't know that…the likelihood of people getting bowel cancer if they've been diagnosed with Lynch syndrome so yea it's good to know endometrial cancer is as high as well" [GP23]*<br>*"Lynch linkage in people's brains is programmed for bowel rather than women's cancers" [GO18]*<br>*"HNPCC—hereditary nonpolyposis colon cancer, and…even just that you know kind of suggest that it was mostly to do with colon cancer than anything else." [GYN13]*<br>*"Oh I don't know…I had good intentions of doing some reading up on it prior to this…but I don't know." [GP24]*<br>*"I'm not sure, it must be higher because one of my patients who had endometrial cane- cancer…sent her off to the geneticist to see if she did have Lynch syndrome." [GP26]*<br>*"I think it (endometrial cancer) rates after colon in terms of risk."[GE27]*<br>*"I'm not a geneticist and I couldn't give you numbers (risk estimates)"[GP6]*

Table S2. *Cont.*

Thematic category	Barriers	Direct quotes
	Lack of adherence to guidelines	*"the national breast and ovarian cancer centre has got some guidelines they're probably more to do with breast cancer than ovary but I'm sure they have some guidelines" [GO2]*<br>*"...it makes someone feels useful because they put out guidelines…it doesn't work" [GE16]*<br>*"in my experience, written guidelines given out to possible referrers don't work because nobody's got time to read them" [GE20]*<br>*"More and more people are coming out with guidelines, I mean, we are getting bombarded with guidelines it's really hard to know exactly what to do" [GP6]*<br>*"there's no strict guideline to say but I don't know that would be actually much more helpful than you know if you see an Amsterdam criteria come up in your history taking" [GO18]*<br>*"where to find these guideline is a problem change of guideline is a problem...and the accessibility and the uh- accountability as well who is going to be responsible if you don't follow the guideline?" [GYN12]*<br>*"if there's been family history that's really been my only guideline" [GP28]*<br>*"I've got my own criteria which is if they have a whole lot of polyps I send it" [GE3]*<br>*"I think that's where things like the Amsterdam business is quite a good little quick ready rule of thumb to think yes no and if there's any doubt just send it to the genetics" [GO18]*<br>*"what would be the criteria that they (clinical genetics services) want to see patients referred …and then guidelines for referral for consideration of Lynch or other syndromes would be useful" [GE25]*

Table S2. *Cont.*

Thematic category	Barriers	Direct quotes
	Negative attitude toward genetic testing	*"potentially opens cans of worms as far as the daughter's insurability is concerned…knowing that she's got the genetic predisposition to cancer doesn't change anything… research has got to lead uh at some stage into action either treatment action or preventative action" [GYN10]*<br>*"there is a limitation on how useful testing is…how is this going to affect this person's life is it going to add quality of life or quantity of life" [GP11]*<br>*"…genetic counselling is something happens after the event so once you've made a diagnosis once you've treated the patient, the patient is in the convalescence period and then you start thinking about well who else could possibly have this." [GYN4]*<br>*"if we go ahead do genetics screening what is the implication for them for their risk also what's the implication for them for getting things like insurance policies"[GP6]*<br>*"knowing this genetic information does have some potentially insurance implications…it has implications for other family members who may or may not want to know"[GO9]*<br>*"this is very time consuming and something that I would not normally do and so then in that situation I would definitely send to a geneticist" [GYN4]*<br>*"it is quite time consuming going through a big family history… chasing down actual diagnosis and is (there) a team set up for that?" [GO18]*<br>*"this takes time to discuss and it takes a lot of time to look at patients to sort of go through that sort of things and our system is of general practice is not set up for that" [GP6]*<br>*"It's not a field that particularly interests me… It's not a field that makes any impact on my personal life nor my professional life directly anyway" [GYN10]*

Table S2. *Cont.*

Thematic category	Barriers	Direct quotes
	Lack of personal and professional experience	*"I haven't come across anyone in my practice—it's a rare thing" [GYN13]*<br>*"We actually are familiar with what to do with them but I guess if you're an isolated practitioner you wouldn't see many of these patients. You'll be lucky to see one every two years, probably, and then you might not quite know what to do with them. I could've imagine that'll be a barrier just like a familiarity...so some sort of education is needed isn't it?" [GYN19]*<br>*"HNPCC is only about 1%–2% so we're talking the very minority of people coming through." [GE3]*<br>*"I think the numbers of people per year you'd see who had something that you think might be Lynch syndrome you can probably count on one hand...or probably a couple of fingers per year" [GE27]*<br>*"I don't know if I've ever seen a case of Lynch syndrome I may or may not I mean it's quite possible that some patient I had in the past has turned out to have Lynch syndrome I mean I just don't know" [GYN10]*<br>*"I haven't come across anyone in my practice it's a rare thing" [GYN4]*
	Uncertain of who or when to refer	*"I think not everyone necessarily understands when you should need to send someone to a geneticist, when you should and when you shouldn't, I think Lynch syndrome is particularly hard..." [GE25]*<br>*"The referral will probably be low because the clinicians aren't picking up the patients who should be referred on" [GYN13]*
	Lack awareness of importance of family history	*"the biggest limiting factor is lack of clinician awareness about the importance of a family pedigree" [GO9]*<br>*"GPs probably need to take a more detailed family history" [GP26]*

Table S2. *Cont.*

Thematic category	Barriers	Direct quotes
**Patient-related factors**	Patients disinterest	*"I'll make the referral whether they take it up or not is another question altogether" [GO18]*<br>*"I can make a referral and patients won't go if they don't want to." [GP6]*<br>*"…my job is I guess to provide the information and then the patient can decide what they want…" [GO9]*<br>*"I've suggested patients to go and they don't want to or they don't see the importance of it" [GE14]*<br>*"Some patients don't want to go there you know they're stressed or you refer them they don't turn up… I guess patients are often quite scared" [MO17]*<br>*"People get worried about cost of genetic testing because it's quite expensive a lot of these tests are expensive…certainly not lacking of trying to get them there…some people just don't want it. I've asked them and they said I don't I'm not interested" [GO18]*<br>*"the cost might be a barrier for the patient" [GP21]*<br>*"That has been brought up by one patient they were worried about it getting the testing done with the uh repercussions for their children" [GO8]*<br>*"people get afraid of what they don't know so when they don't know what exactly will happen to them when they go and what to expect" [GP23]*<br>*"the patient is going to be more interested in their own condition once the diagnosis of cancer is made and wanted to get that treated as soon as possible and I think genetic counselling and all that is probably a secondary issue for the patient themselves"[GYN4]*<br>*"The other part of it is the patient's not seeing it as important..." [GP26]*<br>*"I'm an advocate really I mean if there is a high risk of getting a second malignancy from their mutation I would say this is what I would recommend or and then it's up to the patient to make the choice" [MO5]*
	Lack detailed family history for appropriate referral	*"…no patients know their full family history until they go asking sometimes you got to trigger them to go back to the family and ask for them what the broader family history is" [GE14]*<br>*"…a lot of family history is not always known by patients" [GP28]*

Table S2. *Cont.*

Thematic category	Barriers	Direct quotes
**Organizational-related factors**	Uncertain or perceived long wait time for a genetics appointment or test results	*"there's a much longer wait so again sometimes you've forgotten about it" [GO1]*<br>*"I think the waiting list is several months" [GP11]*<br>*"I don't know how easy it is to get into clinical geneticists maybe some hideous waiting time...of years" [GYN10]*<br>*"My patients would prefer- would want an earlier appointment as opposed to be on the list be on the waiting list… I would think that the waiting list for a geneticists in a public hospital probably be not very short" [GYN13]*<br>*"…don't think it's too long. I think it's only a few weeks or maybe longer. Not sure. Probably varies at different times of the cycle." [GE14]*<br>*"…they have to wait a bit … I'm just familiar with people happy to wait."[GYN19]*<br>*"the test…takes some months to get an answer… it's probable that the waiting time is less in the private system than the public system but I don't know" [GO2]*<br>*"the waiting time to see (private geneticist) is probably shorter" [GE20]*<br>*"I don't know what the waiting time is" [GP23]*<br>*"it does take a while for patients for people to see a geneticist the waiting list is long…public outpatient services is not very good... we have a waiting list... that might act a bit as a road block" [GE25]*<br>*"the barriers is just the referral process to get to see someone at the public hospital" [GP26]*<br>*"we know in the public system it takes them ages to get in there and seen and dealt with" [GYN4]*<br>*"the process takes a while … it might be a month or so before the patients get seen by the genetic services it could be another six to twelve months before the mutation testing results" [MO5]*<br>*"I don't really know…how long that process takes I can't tell you" [GO7]*<br>*"…the waiting time is less in the private system than the public system but I don't know…" [GO2]*

Table S2. *Cont.*

Thematic category	Barriers	Direct quotes
	Unknown cost or assumed high costs of testing	*"It's a significant cost testing as far as everyone goes I mean I believe in the thousands of dollars." [GP6]*<br>*"Lot(s) of it is not covered by Medicare so patients do end up out of pocket so that can be a barrier." [GP11]*<br>*"I don't know what contribution private health insurance would make to the payment. I don't know where the covers are but I think if you've got a reasonable risk of having a mutation I suspect Medicare picks up the cost of your screening for your mutation" [GO2]*<br>*"is there a medical rebate for the patient?" [GP24]*<br>*"well I thought that cost is not an issue with the public sector. Is that right? I don't know about the cost" [GO8]*<br>*"some of the patients that I see you know can barely afford to have the private health insurance and I take it there's a rebate…for genetic counselling. Is that right?" [GYN4]*<br>*"so many barriers in terms of the cost of the thing. you've got to make an appointment with your GPs, you got to pay for that, you got to pay for an appointment with the genetic counsellor, and you've got to pay for the test I mean this is all in private potentially in public you know you don't have to pay for it" [GO1]*

Table S2. *Cont.*

Thematic category	Barriers	Direct quotes
	Unfamiliar with genetic services	*"No I've never referred anyone like that. In fact I never knew that this service exists." [GYN15]*<br>*"I don't know if there are enough of them (geneticists) to provide adequate service or not." [GYN10]*<br>*"When I first came to town I don't know where to send them…I didn't know there was a service until someone said…somehow I'd sent one. Awareness is zero; accessibility is less than zero, because you're not visible. Because you're not…it's not a transparent easy to do service." [GE16]*<br>*"I can't find the cancer care website half the time, I work here, and I'm sitting in it you know so like that's not you know, information distribution in general is an issue" [MO17]*<br>*"I don't know what investigations the genetic people would want…"[GYN19]*<br>*"…knowledge also about the- the way to refer you know where the services are available and how practically to write a referral would also be probably a barrier" [GE20]*<br>*"I could not comment on what the different genetic unit used in other place in terms of you know in terms of how they choose their patients to be tested" [MO17]*<br>*"I don't know what investigations the genetic people would want..."[GO18]*<br>*"…do the geneticists themselves do any testing because I'm not sure about that... are they scientists? are they doctors? what are they?"[GYN4]*<br>*"There's two ways of looking at the genetic health, something to do with fertility and miscarriages, and oh three reasons I suppose, the second thing is general medical condition, if you see somebody's ears look different mom's ears look different face is different will that persist in cardiac abnormality or something" [GYN12]*<br>*"…you can see a GP anywhere pretty much but you can't see a genetic counsellor everywhere." [GO1]*<br>*"The availability of service is important but how anyone would know there's a phone call through the switch would get you to the genetic health. Is the genetic team does enough lecturing…well I think they don't do as much and they should" [GYN12]*

Table S3. Motivators for referral of patients with suspected Lynch syndrome to cancer genetics services.

Thematic category	Motivators	Direct quotes
**Clinician-related factors**	Knowledge of family history and age at diagnosis	*"Women with a strong family history that we see obviously we have to trigger our memory to go… oh yes lots of cancers in that in that family…so that we would then trigger it to get tested" [GO1]*<br><br>*"family member or family history is the biggest trigger to see a genetic counsellor" [GP11]*<br><br>*"I would always take family history... and if I can see that there's any history of increased risk of breast or bowel or endometrial cancer then I would definitely refer to genetic counselling...or genetic testing" [GYN13]*<br><br>*"…family history would be the first indicator" [GE14]*<br>*"I guess a reasonable sort of (family) history… had an endometrial cancer microsatellite instability is positive under 50 then they get referred" [GO18]*<br>*"if there's quite a strong family history we'll just send them off" [GYN19]*<br>*"...in their family history they may say oh their father had colon cancer and their uncle had colon cancer and you know that starts to ring alarm bells" [GO2]*<br>*"...seeing a significant family history...seeing pathological features which concerns us you "...know if we saw a young person some part of their pathology you know matches with a potential um genetic predisposition then we'd refer them through" [MO22]*<br>*"...aren't readily recognized unless there's been family history" [GP28]*<br>*"we're guided by the family history and what the malignancies obviously ovarian where there is a strong hereditary link such as breast and ovarian cancer for example" [MO5]*
	Knowledge of tumour test results	*"If they have mismatch repair (testing) then I'll be more inclined to refer if there's no mismatch repair then the chances are that I'd probably won't refer because it's an isolated case" [MO5]*<br>*"I would give them a referral for that... to get them seen to have the discussion from what I'm aware of what the options are and then consider testing from there. In the mean time I'd also talk to the pathologist and get them to do mismatch repair genes on the pathology for this particular condition" [GO1]*<br>*"Not all services, for example, histopathology services, do the immunohistochemistry stains for the MMR genes so that's a problem." [GE27]*<br>*"I'd look into what tests are available and go ahead and do those and depending on the results then I'd refer them on to a genetic counsellor." [GYN4]*<br>*"We do mismatch repair genes on pathology and then … I'd say we're probably initiate the referral to genetics before the patients on the basis of mismatch repair genes. that will means mismatch repair genes on the pathology that's suggestive of a Lynch then you know I'm more than happy to send her off to genetics" [GO7]*<br>*"We do immunohistochemistry as a sort of a screening tool and then based on that we can refer them on for formal molecular genetic testing."[GO9]*<br>*"if a patient has developed cancer at a very young age…immunohistochemical studying for mismatch repair genes in particular endometrial cancer can be a guide for further evaluation."[GO9]*

Table S3. *Cont.*

Thematic category	Motivators	Direct quotes
	To improve patient diagnosis, treatment and clinical management	*"if a genes is being identified in your family and you can be tested in one way or the other then I think that opportunity needs to be given to everyone" [GP11]*<br>*"as far as the patients are concerned the nitty grit has got to be either prevention or treatment, ideally prevention of their cancer" [GYN10]*<br>*"It's going to help you to some degree because you've got this problem and therefore you can protect yourself against other cancers... it will help your other family members decide whether they want to be tested" [GO1]*<br>*"Genetic testing is very useful … to firm up the diagnosis in the proband, because that might influence what screening you and treatment you recommend for that individual proband… furthermore, that makes a difference to what you recommend to the family…" [GE20]*
**Patient-related factors**	Patients requests	*"I'll be very happy to refer people to genetic counselling if that's what they want" [GYN 10]*<br>*"If they asked me to refer, I've got no concerns about sending them." [GYN4]*<br>*"most of the time the question of genetics comes up is usually something that I raised with them" [GO2]*<br>*"I think that would be slightly more common for us to suggest it (referral) probably. Often those women would have concerns for their children and they want to be tested for that reason." [GP11]*<br>*"I'd be very rarely asked that question (to be referred for genetic services)." [GE14]*<br>*"I can't even think I've had anyone had said that to me to be perfectly honest, I think genetic counselling and the role of genetics is not well known to patients, so that would (be) unusual it would be much more common for me to suggest that I send them to genetics rather than the other way around" [GE25]*<br>*"I've never had a patient asked me for a referral to a genetic counsellor." [GP24]*<br>*"No the patient usually does not request that. It's up to the practitioner to decide." [GYN15]*<br>*"If the patient's motivated about their health and they've read about it and they think 'oh my family history' they're going to ask their GP" [GO1]*<br>*"More likely if they've had a cancer diagnosis then they will (ask for referral to a geneticist)" [GP26]*<br>*"I think it was an older brother ... been diagnosed with cancer and this time my patient's only in her late 30s and she came and she said look you know someone in my family's been diagnosed with Lynch syndrome and I need to get the genetic test." [GP23]*<br>*"...typically when they have daughters... you know, if they got daughters, they want to get it all done for their daughters" [GO18]*

Table S3. *Cont.*

Thematic category	Motivators	Direct quotes
**Organisational factors**	Practical information about genetic services (e.g. the availability and cost of testing, turnaround time)	*"In general practice really you just want the practical stuff the stuff that works for people and I guess accurate information on the availability and cost of testing is also useful because people who often read stuff in the media will think or come and say oh look, this test is available and I can be tested for this gene" [GP11]*<br>*"...it would be helpful to know how many geneticists are available in Brisbane." [GP24]*<br>*"we often get very good information about their surgery their chemotherapy their radiotherapy* etc. *but we don't get the good information about what has been discussed as far as their risk of other cancers and their family risk and those sort of things so I don't get a lot of that back."[GP6]*<br>*"I guess knowing who is available and you know what their fees are…to think about perhaps referring these patients" [GP24]*<br>*"I guess a succinct sort of saying a little bit education identifying well who we should be considering for referral that's the best way to go and then looking at well how do I do the referral, that's, you know, what's the pathway. That's the most important thing" [GP6]*<br>*"Really don't want to someone to be waiting for 8 months for an appointment so it would be much nicer if you can look up and see what their waiting times are." [GP23]*<br>*"You need a one stop shop that says genetic counselling, one call number, one person who doesn't do anything just says that kind of counselling goes there, that kind of counselling goes there, just give me your details, I'll pass it on" [GE16]*<br>*"what would be the criteria that they want to see patients referred but also a very brief dot points of the things that they can offer… advice with regards to life insurance and family planning… the key roles of clinical geneticists and then guidelines for referral for consideration of Lynch or other syndromes would be useful" [GE25]*<br>*"if we can have the molecular genetic testing done faster, and if we have a bigger bet- and better library of um mutations again that would be useful" [GO9]*<br>*"...a hand out that told us what particular investigations you wanted prior to the patient attending" [GO2]*<br>*"...what genetics does and how genetics operates in Queensland is probably (good)" [GE20]*<br>*"I think more information needs to be out there about the genetic service... Making the public more aware or the doctors more aware... or how to do a referral" [GO8]*<br>*"How long does the lab keep the tissue, for example? So, if a patient goes and have surgery and they're in the hospital for a week or whatever and then they come home and then they might come back to the GP 2 weeks later, if the- the surgeon hasn't actually requested that on their pathology request is the tissue still in the lab for me then to add that as a request?" [GP24]*<br>*"it would be handy to know what the eligibility of having some of those tests would be" [GP21]*<br>*"I think the referral pathway is clear but it's probably it would be helpful to have some clarification on the level of suspicion that's needed for referring on" [GP11]*<br>*"Referral pathway is always good for most of the service. The problem with referral pathway if it's not enough education from the service provider." [GYN12]*<br>*"a hand out that told us what particular investigations you wanted prior to the patient attending" [GYN19]*<br>*"Should give the patients the brief explanation of what medical genetics is about and who to contact and how to you know how to make an appointment." [GO2]*<br>*"...maybe you should be distributing some sort of paper work that you know work sheet that we can hand to patients and say now this is probably what's going to happen because in our own processes we have consents...any procedure we do and those consents include a full list of the procedures what's going to be done and the possible complications even quite unusual bizarre and infrequent complications we have to inform patients so... something like that would be valuable" [GYN19]*

Table S3. *Cont.*

Thematic category	Motivators	Direct quotes
	Specific criteria or guidelines for referral	*"Referral guidelines are always helpful guidelines are good clinical guidelines that sort of things is good. I don't know I haven't seen one for Lynch but I mean there are for breast and ovarian" [GP11]*<br>*"Guidelines would be good. we have Amsterdam criteria but it'd be nice if there was Australia wide guidelines—that would be ideal" [GO7]*<br>*"I think guidelines and pathways for referral will be more important…having the pathways and making sure everyone's aware how to do that easily whether you do it on paper or online doesn't matter" [GO8]*<br>*"I think things are changing all the time and guidelines on referrals for patients to the service would be you know would be you know a reasonable thing…a spread sheet that clinicians who don't deal with this regularly could look at and say oh I've this Bethesda criteria I'll refer them on… I do think that providing some kind of uh referral guideline would be sensible" [GE25]*<br>*"Easy guideline as to who is it... who we should consider referral for and probably just make having easy pathways for referral to a geneticist" [GP26]*<br>*"I think having clearer guidelines from genetics might be helpful such as when to refer... you know it'll be ideal if they have them on a board in the multidisciplinary room" [MO17]*<br>*"…guidelines for referral for consideration of Lynch or other syndromes would be useful" [GE25]*<br>*"it'll be good if (a) we did have some guidelines and (b) if there was a screening test that you could sort of say look you know even though you have got only family history we don't know anything else but you know we can always do test a, b, c and see if you know if there is a component to it then referral to a geneticist" [GP28]*<br>*"GPs could know more about it and have some guidelines" [GP26]*
	Increased collaboration with genetics specialist	*"a multidisciplinary approach is good because then you know surgeons and medical oncologists and radiation oncologists and pathologists can bound ideas and often you know I'm not all knowing but you learn as you go along. So, yea, I think, yea, I think you know your decision is made at a multidisciplinary setting then everyone would be comfortable you know that you don't have to take the sole responsibility of doing it" [MO5]*<br>*"we sent them for second opinion for the tumour board meeting and then waiting for reply…so it's always good to have team consultation…there's a bridge always...we have to have…share the responsibility… I think it's mutual responsibility...not only it's important and that is multi-responsibility." [GYN12]*<br>*"It would help even got we have constant changes in registrars you know I mean those sort of things people coming through I mean it's excellent training for them to be aware of another facet you know because we're not the same medical oncologist and genetic people but you know we complement each other and I think working together would be a good thing." [MO17]*<br>*"The middle ground is where the patients being dumped. They are being dumped...and that you need that sort of cooperative approach" [GE16]*<br>*"if you start sending patients to genetics and you start getting letters back and you start therefore to learn more about how the process works and...oh, that one I sent…they didn't say there was anything we can do to help, but this one, you know there's all these good outcomes happen from it. That's the sort of learning that it's probably the most effective and then those doctors are more likely to keep referring and to fine tune their referrals." [GE20]*<br>*"…improve public awareness of it (genetic services)" [GYN10]*<br>*"…maybe there should be more promotion at the level of clinicians…maybe more contribution to the grand rounds and talks... a regular flyer or newsletter..." [GYN12]*

Table S3. *Cont.*

Thematic category	Motivators	Direct quotes
	Prompts or triggers for referral	*"having a list of associated syndromes ... sort of as red flags to think about perhaps referring these patients" [GP24]*<br>*"generalists who don't deal with cancer a lot, they need to be continually reminded" [GYN4]*<br>*"I think there's an online tool that you can use to actually work out the BRCA risk but I don't know if they have…if the tool was used was validated for HNPCC or Lynch… I think a tool which is sensitive and specific would be good if we could then plug a few parameters in and then tells you the risk of getting you know colon or breast or whatever cancer uh having an online tool would be quite useful" [MO5]*<br>*"I don't think there are barriers; they're just not… people (not) thinking to do it. That might be more not such a barrier but a lack of a trigger to that problem you can say it that way… It's always helpful for the patients to come back with the complete map of their family when there's a suspicion there. Some will do that. They just map it out and send it back to you" [GE14]*<br>*"You can put a little sort of sticker on their desk or something that flashes up on the screen (to remind them)..." [GE14]*<br>*"I don't know whether in some ways (a) clinical software there could be some way that you can flag everyone that's got a family history of breast cancer for instance and then pull them up and then sort of send them a brochure on genetics in breast cancer...that...might be one way to do it" [GP21]*<br>*"what we're booking should be on the Internet… might be appropriate for you to go the weekend you know you go to the place you want and that's taken you to the next place and then we can have a look and see… Someone would do it would make so much difference in general practice just to have a little specialty booking" [GP23]*
	Ease of access	*"I ring genetic counselling at the (public hospital) and there (is) usually a person in that department...who's looking after that. All I need (is) a phone call, I ring directly upstairs or ring the switch to put me through" [GYN12]*<br>*"I just ring them and say well you know I need the patients to be on the list and (they) say okay well this is the date and time the patient can come and see me." [GYN13]*<br>*I think I get a fairly good service just because I'm on the same campus and I know these people [GE25]*<br>*"It's not a big deal for me it's just a phone call" [GE3]*<br>*"In private world is just so much easy, you can just ring the geneticist will start making an appointment really so...and knowing the appointment and just liaise with them regarding triaging as well so...in private world is just so much easier" [GYN13]*
	Continuing education for clinicians	*"more education for you know for everybody and if GPs are educated that these conditions exist because the GP would often see the whole family" [GO1]*<br>*"Grand rounds and lectures for education…for genetic syndrome (to) make people more aware" [GYN12]*<br>*"just ongoing education of clinicians is really the mainstay other thing because it is the clinician if they have the knowledge they are the ones who are then going to be able to pick the patients, explain to the patient the importance of the test and then referred them on to the genetic testing so I think education again of the clinicians is one of the sole factors it's going to make a huge difference to the referrals yes." [GYN13]*<br>*"...increase knowledge of the diseases …is probably (good)" [GE20]*

Table S3. *Cont.*

Thematic category	Motivators	Direct quotes
	Better follow-up care or referral pathway	*"more important than the referral to the geneticists is sometimes the kind of coordination of the you know prevention and management" [MO22]*<br>*"one of the major roles is having highly qualified nurses who support the registry and chase up patients when they're when they're due for you know screening into tests" [GE25]*<br>*"to have (a) central database… someone appointed to kindly follow up on those results to say did someone get referred or did that turn out to be Lynch syndrome" [GE27] "The system should be set up for the patient's convenience...and should be set up for the convenience of the referring doctor" [GO2]*<br>*"...you need awareness, repetition, transparency in the process—it needs to be an easy process…access, visibility, transparency and immediacy. Immediacy is basically saying ... ring this number give them your details and off you go" [GE16]*

© 2014 by the authors; licensee MDPI, Basel, Switzerland. This article is an open access article distributed under the terms and conditions of the Creative Commons Attribution license (http://creativecommons.org/licenses/by/3.0/).
